# Review on the use of sexually dimorphic characters in the taxonomy of Diabroticites (Galerucinae, Luperini, Diabroticina)^1^

**DOI:** 10.3897/zookeys.332.4931

**Published:** 2013-09-19

**Authors:** Laura Rocha Prado

**Affiliations:** 1 Museu de Zoologia da Universidade de São Paulo, Av. Nazaré, 481, CEP 04263–000, São Paulo-SP, Brazil

**Keywords:** Taxonomy of Coleoptera, rootworms, review, leaf-beetles

## Abstract

Sexual dimorphism occurs frequently in Chrysomelidae Latreille, 1802 and is particularly variable in subfamily Galerucinae Latreille, 1802. This diversity has been early noted by authors a potential source of taxonomic characters. The section Diabroticites (Luperini Gistel, 1848) is one of the largest assemblies of chrysomelid genera with currently 823 valid species in 17 genera (12 based on dimorphic characteristics), being most diverse in the neotropical region. Apart from a revision work on the type specimens for the section, there are no general taxonomic studies for this group. The occurrence of sexually dimorphic characteristics in the section Diabroticites is revised and their practical taxonomic relevance evaluated. A total of 240 species was studied (145 species with males available), representing 15 out of the 17 genera included in Diabroticites. The analysis of characters was based on the study of specimens in south-american collections, literature and the aid of photos in online databases. Sexual dimorphism occurred in most species analyzed. Dimorphic features were divided in general (i. e., occur in higher taxa) and special characters (those that support the definition of species and genera). Special dimorphism was observed in every tagma, and most modifications occur in antennae. Characters used as diagnostic of genera often do not correspond to the modifications present in species included in them. Many modifications were considered by earlier authors as a single character, probably due to vague definitions. Most generic definitions are, therefore, inaccurate. The study of morphology and the homology assessment of characters are needed to increase understanding of the genera in Diabroticites.

## Introduction

Sexual dimorphism has always been a subject of great curiosity amongst naturalists. Since Darwin’s suggestion of his Theory of Sexual Selection, many explanations have been proposed, and several have been successfully tested, for the existence of often peculiar modifications in males. Even though most beetles lack conspicuous sexual dimorphism (Kawano 2006), there are striking examples in all major Coleoptera taxonomic groups (Eberhard 2009). Most sexually dimorphic characters in beetles are described by strongly positive allometries (Kawano 2006), and also modifications found in antennae, tarsi, posterior legs and ventrites (Crowson 1981). Other less common characteristics include the reduction of the wings (Thayer 1992) and the presence of luminescent (Branham and Wenzel 2003) or stridulatory organs (Jansson and Selander 1977).

In Chrysomelidae Latreille, 1802, sexual dimorphism is thought to occur more frequently at the species level (Jolivet and Verma 2002). Common dimorphic features that are the body size (with females usually bigger than males) and the modification of tarsi, usually related to greater adhesion of males to the females’ dorsal surface during copulation (Jolivet and Verma 2002, Hammack and French 2007, Voigt et al. 2008, Nardi et al. 2012).

Within the subfamily Galerucinae Latreille, 1802, sexual dimorphism is particularly variable, as Mohamedsaid and Furth (2011) have illustrated and summarized. This diversity has been early noted by authors as a potential source of taxonomic characters. As Horn (1893) pointed out, many taxonomic issues related to this group could be resolved with the aid of “sexual peculiarities”, which could be a useful guide for understanding the relationships between species. Blake (1958) also stated that the use of such characteristics could help the delimitation of genera in problematic groups such as the tribe Luperini Gistel, 1848.

The section Diabroticites Chapuis, 1875 (Luperini) is one of the largest assemblies of chrysomelid genera with over 900 recorded names in 17 genera (12 based on dimorphic characteristics), being most diverse in the neotropical region. Apart from a revision work on the type specimens for the section (Smith and Lawrence 1967), there are no general taxonomic studies for this group. The most recent catalogue mentions 793 species (Wilcox 1972), but a review of the subsequent literature reveals that the group has currently 823 valid species. Table 1 presents an overview on the current composition of Diabroticites. Prior to 1906, Diabroticites included *Diabrotica* Chevrolat, 1837 (almost half of the total species in the section), and three other genera, which were all monotypic: *Ensiforma* Jacoby, 1876, *Pseudodiabrotica* Jacoby, 1892, and *Paratriarius* Schaeffer, 1906. Barber (1947) was the first to investigate male genital characters to understand the relationships within the group and described two more genera in the section, *Acalymma* Barber, 1947 and *Amphelasma* Barber, 1947. In the subsequent years, the remaining 11 genera were erected, eight of which based on species formerly included in *Diabrotica* (*Anisobrotica* Bechyné & Bechyné, 1969, *Aristobrotica* Bechyné, 1956, *Buckibrotica* Bechyné & Bechyné, 1969, *Cochabamba* Bechyné, 1955, *Cornubrotica* Bechyné & Bechyné, 1969, *Gynandrobrotica* Bechyné, 1955, *Synbrotica* Bechyné, 1956, and *Paranapiacaba* Bechyné, 1958), and most supported primarily on dimorphic features (Table 1). Surprisingly enough, only one genus has a detailed description of genital characters (the most recent genus, *Platybrotica* Cabrera & Cabrera Walsh, 2004).

**Table 1.** Overview on the composition of genera of Diabroticites. The total number of analyzed species includes specimens in collections and online type-specimens in MCZ database.

**Genus**	**Dimorphism as diagnostic**	**Number of species in original description**	**Current number of species**	**Number of analysed species (% of genus total)**
*Acalymma* Barber, 1947		6	72	28 (38%)
*Amphelasma* Barber, 1947		5	11	2 (18%)
*Anisobrotica* Bechyné & Bechyné, 1969	X	1	5	5 (100%)
*Aristobrotica* Bechyné, 1956	X	10	17	3 (17%)
*Buckibrotica* Bechyné & Bechyné, 1969	X	1	1	1 (100%)
*Cochabamba* Bechyné, 1955		4	10	10 (100%)
*Cornubrotica* Bechyné & Bechyné, 1969	X	1	2	2 (100%)
*Diabrotica* Chevrolat, 1837		103	360	114 (31%)
*Ensiforma* Jacoby, 1876	X	1	9	3 (33%)
*Gynandrobrotica* Bechyné, 1955	X	23	32	4 (12%)
*Isotes* Weise, 1922 (= *Synbrotica* Bechyné, 1956)	X	1(51)	182	38 (20%)
*Palmaria* Bechyné, 1956	X	1	1	-
*Paranapiacaba* Bechyné, 1958	X	16	59	14 (23%)
*Paratriarius* Schaeffer, 1906	X	1	51	11 (21%)
*Platybrotica* Cabrera & Cabrera Walsh, 2004	X	1	1	1 (100%)
*Pseudodiabrotica* Jacoby, 1892	X	1	1	-
*Zischkaita* Bechyné, 1956		1	9	4 (44%)
Total	12	177	823	240 (28%)

About 80% of the diabroticites species have been described prior to 1895, mainly by Joseph Sugar Baly, Charles J. Gahan and Martin Jacoby (Smith and Lawrence 1967). Most of those descriptions lack detailed morphological information, and usually depict characters relative to color pattern and, sometimes, punctuation. For many of the genera this is also true, with internal characters being almost completely ignored. General morphology has also been vaguely treated, described usually without any aid of illustrations whatsoever. Such scarcity of information and the apparent uniformity in morphology of some diabroticites resulted in a difficult taxonomic scenario.

The purpose of this study is to summarize the occurrence of sexually dimorphic characteristics in the section Diabroticites, as well as to review these characters chosen by earlier authors to support their definitions of genera and evaluate their practical taxonomic relevance. Systematic research is being conducted on Diabroticites by the author, and the first results are reported here.

## Methods

The analysis of characters was based on the study of specimens, literature (original descriptions and revision works, when available) and the aid of photos in online databases. A total of 240 species was studied, representing 15 out of the 17 genera included in Diabroticites.

Specimens were obtained from south-american collections listed in Table 2, always in comparison to original descriptions – and many types were available. Out of the total species available, only 145 species had males available or known. These taxa were listed in Appendix. The specimens were examined and illustrated using a Zeiss Discovery.V8 stereomicroscope with a camera lucida attached. Final art was done in Adobe Illustrator®. Photographs were taken using a Leica M205C stereomicroscope with an attached magnifying lens and Leica DFC 295 video camera. Image combination was performed with Leica Application Suite V3.6.0, and subsequent edition was done in Adobe Photoshop®.

**Table 2.** Institutions that provided specimens for the study.

**Acronym**	**Name**	**City**	**Country**	**Curator**
CEAH	Coleção Entomológica Adolph Hempel, Instituto Biológico	São Paulo	Brazil	Sérgio Ide
INPA	Coleção Sistemática de Entomologia, Instituto Nacional de Pesquisas da Amazônia	Manaus	Brazil	Augusto Henriques
FIOC	Fundação Instituto Oswaldo Cruz	Rio de Janeiro	Brazil	Jane Costa von Sydow
IACC	Instituto Agronômico de Campinas	Campinas	Brazil	Édson Possidônio Teixeira
MGAP	Museu Anchieta	Porto Alegre	Brazil	Fernando Meyer
MCNZ	Museu de Ciências Naturais da Fundação Zoo-Botânica do Rio Grande do Sul	Porto Alegre	Brazil	Maria Helena Galileo
DZUP	Coleção de Entomologia Padre Jesus Moure, Universidade Federal do Paraná	Curitiba	Brazil	Lúcia Massuti de Almeida
MZSP	Museu de Zoologia da Universidade de São Paulo	São Paulo	Brazil	Sônia Casari
MNRJ	Museu Nacional, Universidade Federal do Rio de Janeiro	Rio de Janeiro	Brazil	Marcela Monné
MPEG	Museu Paraense Emilio Goeldi	Belém	Brazil	Orlando Tobias Silveira
UFVB	Museu Regional de Entomologia da Universidade Federal de Viçosa	Viçosa	Brazil	Paulo Sérgio Fiuza Ferreira
MLPA	Universidad Nacional de La Plata, Museo de la Plata	La Plata	Argentina	Nora Cabrera

Most taxonomic literature available for Diabroticites was reviewed. The original descriptions of monotypic genera *Palmaria* Bechyné, 1956, and *Pseudodiabrotica*, known only for their type-specimens, which could not be loaned, were the only source of characters for comparison. Revisionary works were available only for genera *Acalymma* (in part) (Munroe and Smith 1980, Cabrera 1999, Cabrera and Durante 2003), *Diabrotica* (in part) (Marques 1941, Christensen 1943, Krysan and Smith 1987, Cabrera 2000a, Cabrera 2000b) and *Synbrotica* (in part) (Cabrera 1995).

Due to the peculiarity of genus *Isotes* Weise, 1922, which was described based on a single species later found to be a senior synonym of the type-species of genus *Synbrotica* (at that time with over 100 species), the original description of the latter was also included in the analysis for comparative purposes. As a reference to their original descriptions, both names will be used interchangeably throughout the text, even though *Isotes* is the current valid name.

Characters mentioned in original descriptions and other taxonomic works, when available, were compiled and later compared to specimens. Those characters were then redefined, in order to fulfill uniform homology criteria. The broader studies of Mohamedsaid and Furth (2011) and Mohamedsaid (2004) were used for character comparison with other taxonomic groups.

Also, some taxa that had not enough specimens available in south-american collections were studied by the analysis of photos of type specimens available in the Museum of Comparative Zoology online Type Database, Harvard University, Cambridge, USA.

## Results and discussion

Out of the 17 genera that comprise section Diabroticites, 12 have sexually dimorphic features as diagnostic characters mentioned in original descriptions by nine different authors (15 papers) in a period of more than 150 years (Table 1). Most genera were established based on a single or on few species, further taxa being later added, frequently not by their original authors. This information is relevant when checking whether the initial concept proposed for the genus was maintained or not.

Most species studied have some kind of sexual dimorphism. Those modifications were categorized in two distinct groups, concerning its level of taxonomic comprehension: general and special dimorphism.

### General dimorphism

Characters that are referred to as of general comprehension are those proposed several times in the literature as being important to species definition, but were actually found to be more generalized, i. e., they in fact occur in higher taxa. General characters are found in all, or most, male of diabroticites analyzed, and might also support the definition of larger taxonomic groups: **1)** Smaller body size: considered general for Chrysomelidae, observed for most diabroticites; **2)** Bigger eyes (relative to the total size of the head): cited often in species descriptions, but actually observed in most diabroticites; **3)** Tarsal adhesive disks: structures present in most Chrysomelidae, with variation found among subfamilies and often among tribes, regarding the number of legs in which they occur and the proportion of the dorsal surface that they occupy (Stork 1980). For Diabroticites, the adhesive disks are present at the first tarsomere in pro- and mesothoracic legs; **4)** Emargination on the posterior margin of ventrite V: this characters sometimes supports the definition of the subfamily Galerucinae, and its shape can be diagnostic of tribes, being rounded in Luperini and Galerucini, for example (Bechyné and Bechyné 1962, Wilcox 1965). In diabroticites this emargination is, usually, round; and **5)** Prothoracic tibiae with continuous apex, without spurs: probably constant in the whole tribe (Wilcox 1965).

### Special dimorphism

Special characters are those used to describe lower taxonomic ranks, i. e., genera and species, and have been or not mentioned as diagnostic features in original descriptions.

With the exception of two genera, *Amphelasma* Barber, 1947 and *Cochabamba* all genera in Diabroticites are represented with special dimorphic characters. Of all species analyzed, only 47 were found to have special dimorphic characteristics. This is interesting, since the original definitions of diabroticites genera were usually based in sexual dimorphism. In accordance with the pattern observed for the subfamily (Mohamedsaid and Furth 2011), special dimorphism was observed in every tagma in the analyzed taxa of Diabroticites (Table 3).

**Table 3.** Location of diagnostic, sexually dimorphic characters mentioned in original descriptions.

**Genus**	**Antennae**	**Legs**	**Head (except antennae)**	**Elytra**
*Anisobrotica* Bechyné & Bechyné	X	X		
*Aristobrotica* Bechyné	X	X		
*Paranapiacaba* Bechyné	X	X		
*Buckibrotica* Bechyné & Bechyné	X			
*Cornubrotica* Bechyné & Bechyné	X			
*Ensiforma* Jacoby	X			
*Paratriarius* Schaeffer	X			
*Platybrotica* Cabrera & Cabrera Walsh	X			
*Isotes* Weise (ex *Synbrotica* Bechyné)		X*		
*Palmaria* Bechyné		X		
*Gynandrobrotica* Bechyné			X	
*Pseudodiabrotica* Jacoby				X

*: present in the original description of *Synbrotica*.

Out of the 12 genera recognized by dimorphic characters, 4 are monotypic (*Buckibrotica*, *Palmaria*, *Platybrotica* and *Pseudodiabrotica*). The diagnostic characters and its validity will be discussed for each tagma.

### Head

Most of the dimorphic characters were found in the head, mainly in the antennae, a proportion which agrees with the general pattern observed in the subfamily (Mohamedsaid and Furth 2011).

**Figure 1.**
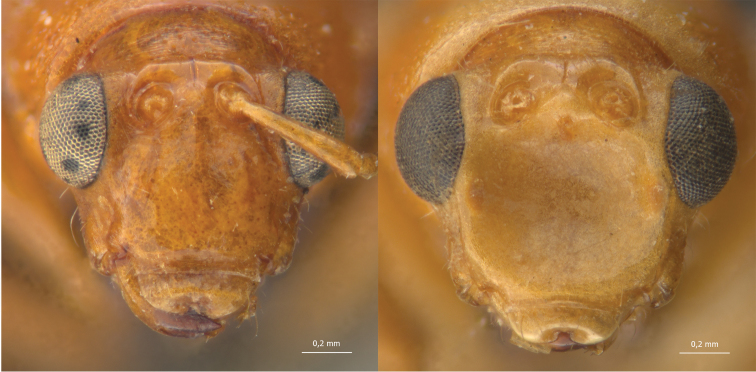
*Gynandrobrotica caviceps* (Baly, 1889), head in frontal view (female, left, male, right).

**Figure 2.**
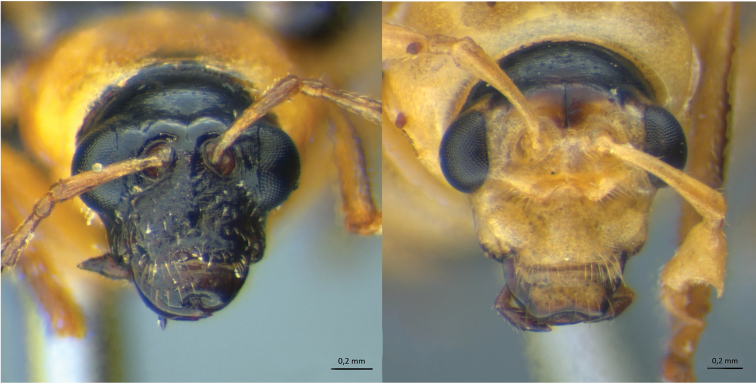
*Cerotoma variegata* (Fabricius, 1792) head in frontal view (female, left, male, right).

*Gynandrobrotica* was described as showing an “excavated clypeus”, without any further details or illustrations. All four species studied have the same kind of modification, which is better described as the frons being elongated and with a shallow, smooth, round concavity (Figure 1), accompanied by sparse, large punctuation. It is interesting to compare this character with the differently excavated type of frons found in species of *Cerotoma* Chevrolat, 1837 (Figure 2), *Eucerotoma* Laboissiere, 1939 and *Neobrotica* Jacoby, 1887 – all of which are usually placed in sister-section Cerotomites Chapuis, 1875. *Gynandrobrotica* has been suggested to be more related to these taxa in some phylogenetic analyses (Eben and Monteros 2004, Gillespie et al. 2008). Other characteristics common to these genera, such as the small eyes, the elongated frons and different shapes of antennomeres I-III should be further studied in order to verify if they are indeed homologues.

**Figure 3.**
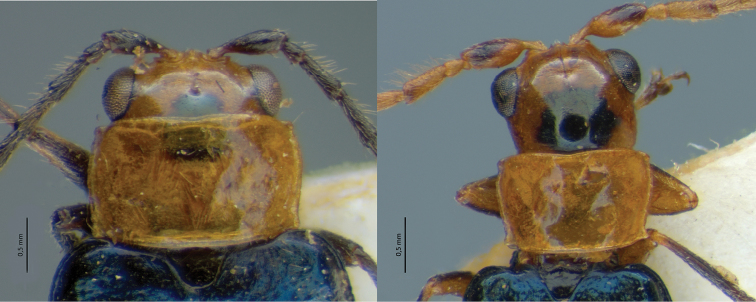
*Isotes onira* (Bechyné & Bechyné, 1961), dorsal view, detail of pronotum and head (female, left, male, right).

There are also three other species with distinctive head features in the male: *Isotes onira* (Bechyné & Bechyné), 1961 has an enlarged head from the vertex up to the antennal insertions – Figure 3), *Diabrotica serroazulensis* Bechyné & Bechyné, 1962 has an enlarged labrum, which is as large as half of the frontal length, and *Acalymma cornutum* (Baly, 1886), has a deep cleft in the frons, with lateral projections, and also a labrum with an acute anterior projection that reaches the frons (illustrated in Munroe and Smith 1980).

### Antennae

**Figure 4.**
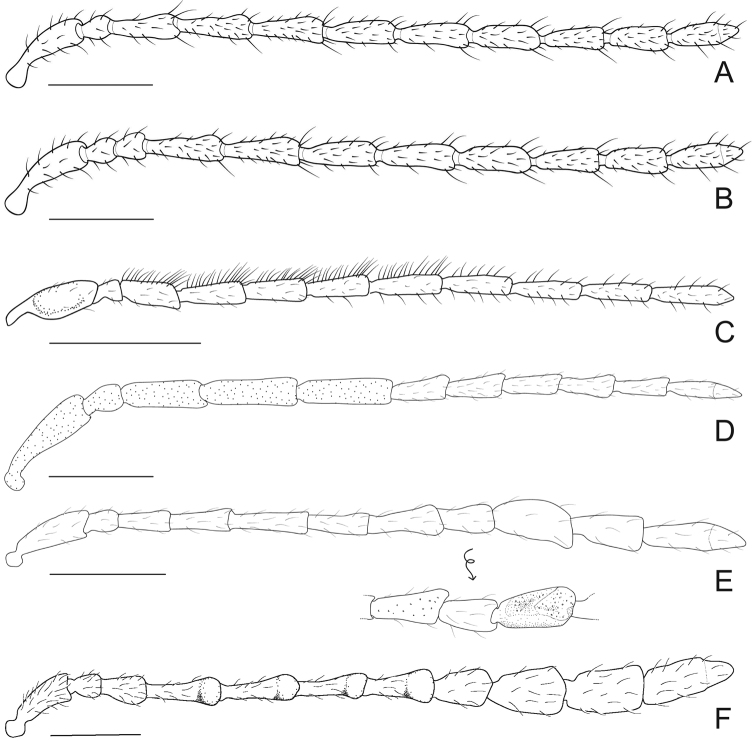
Male modified antennae in lateral view **A** *Isotes borrei* (Baly, 1889) **B** *Cochabamba marginata* (Ha-rold, 1875) **C** *Isotes onira* (Bechyné & Bechyné, 1961) **D** *Aristobrotica angulicollis* (Erichson 1878) **E** *Buckibrotica cinctipennis* (Baly, 1886) (detail in ventral view) **F** *Ensiforma caerulea* Jacoby, 1876. Scale bar = 1 mm.

Galerucines commonly display filiform antennae, which can show numerous dimorphic variations (Jolivet and Verma 2002, Mohamedsaid 2004). This is also true for Diabroticites, with the main antenna type being filiform and antenommeres mostly subequal in size and shape (Figure 4A).

Non-dimorphic modifications are often related to the length of some antennomeres and sometimes support generic definitions. For instance, the two largest genera in Diabroticites, *Diabrotica* and *Synbrotica* are essentially distinguished by the length of antennomere III, described as being subequal to II in the first (same as observed in genus *Cochabamba* in Figure 4B), and subequal to IV (that is, almost twice as longer as II, as in Figure 4C) in the latter (Bechyné 1956).

Eight genera have diagnostic characters based in their dimorphism of antennae (listed in Table 3). Examples of modified antennae are showed in Figure 4C–F. Because of their variability, dimorphic antennae are the most used structure in descriptions, but their modifications have been scarcely detailed. As a result, there are different genera proposed on characters depicted in sentences such as “apical antennomeres modified” (*Cornubrotica*), “antennomeres VII and IX of complicated shape” (*Buckibrotica* – Figure 4E), “antennomeres V-VII modified” (*Paratriarius*).

The absence of unified criteria in the understanding of what a “modified” antennomere is has lead, several times, to the establishment of artificial grouping of species, simply because a single “aberrant” antennomere can display an assembly of four different aspects of its morphology. Modifications include change in (in quoting marks, expressions used on original descriptions): **length** – antennomeres considered “elongated” or “shortened” when compared to the usually fixed antennomeres I and III; **width** – antennomeres described as “swollen”, “inflated” (homogeneous modification), “distally expanded” (heterogeneous modification), and dorsoventrally “flattened”; **shape** – those described as having “lateral projections” or “ventral excavations”; **structure** – antennomeres with “rough punctuation” and different amounts of hairs/sensillae. As variations observed in the species do not always correspond to the diagnosis defined for genera they have been included in, these characteristics do not provide an accurate guide to the identification of taxa in Diabroticites.

**Figure 5.**
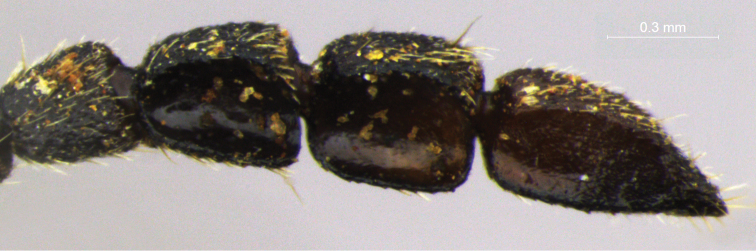
*Anisobrotica donckieri* (Baly, 1889), detail of ventral surface of apical antennomeres, male.

For the non-monotypical genera based on antennal dimorphic features, most original definitions do not correspond to their actual characters. In *Anisobrotica*, for example, the “widened” apical antennomeres do not always appear – *Anisobrotica binisculpta* Bechyné & Bechyné, 1969 only has in common with the other taxa the excavation present in glabrous ventral surface of antennomeres IX-XI (such as excavations observed in apical antennomeres of *Anisobrotica donckieri* (Baly, 1889) in Figure 5). The same happens with *Paratriarius*, which includes several species that do not show “modified antennomeres V-VII” present in type-species *Paratriarius dorsata* (Say, 1824) (illustrated in Wilcox 1965) such as *Paratriarius batesi* (Baly, 1859), *Paratriarius falvolimbata* (Erichson, 1847), *Paratriarius verrucosa* (Jacoby, 1880), *Paratriarius alternans* (Weise, 1916), *Paratriarius nigrotibialis* (Bowditch, 1911), *Paratriarius castanea* (Bowditch, 1911), and also other four species studied by Mohamedsaid and Furth (2011). Instead, these taxa show antennae very similar to the general pattern seen in *Diabrotica*. The two species included in *Cornubrotica* do not show identical antennomeres VIII and IX, although both always have ventral excavations (illustrated in Bechyné and Bechyné 1969 and Moura 2005). In *Paranapiacaba*, the antennal character chosen was, unfortunately, a general one: male antennomeres III-XI uniformly “thickened” (in contrast with slightly slender antennae of females). Nevertheless, antennae do seem to vary uniformly in one genus. In *Aristobrotica*, the pattern of antennomeres III-V “thickened” is constantly repeated, followed by an unmentioned presence of larger punctuation (Figure 4D).

Although Maulik (1936) suggested that, for indo-asian galerucines, the basal antennomeres are more frequently the altered ones, a result that has been corroborated by Mohamedsaid (2004), that feature does not apply to diabroticites analyzed. Also, no obvious topological pattern is seen in the variation of antennomeres (Table 4).

**Table 4.** Selected diabroticites species representing antennal dimorphic variation. Grey cells indicate modification in the antennomere.

**Taxon/antennomere**	**I**	**II**	**III**	**IV**	**V**	**VI**	**VII**	**VIII**	**IX**	**X**	**XI**
*Isotes onira* (Bechyné & Bechyné, 1961)											
*Ensiforma chiquitoensis* (Bechyné, 1958)											
*Aristobrotica angulicollis* (Erichson, 1878)											
*Isotes simplicipennis* (Jacoby, 1889)											
*Ensiforma asteria* (Bechyné & Bechyné, 1962)											
*Paratriarius dorsata* (Say, 1824)											
*Isotes callanga* (Bechyné, 1956)											
*Platybrotica misionensis* Cabrera & Cabrera Walsh, 2004											
*Buckibrotica cinctipennis* (Baly, 1886)											
*Cornubrotica dilaticornis* (Baly, 1879)											
*Diabrotica samouella* Bechyné, 1956											
*Anisobrotica donckieri* (Baly, 1889)											

The number of modified antennomeres oscillated between 1 to 6. Antennomere II was recorded as dimorphic only in species of *Aristobrotica*, such as *Aristobrotica angulicollis* (Erichson, 1878) (Figure 4D) and in *Isotes onira* (Figure 4C). Few modifications occur in antennomeres II and XI. The most affected are antennomeres V to IX. However, there is no indication of an explicit dependency of occurrence between any pair of modified antennomeres. This is the opposite of what has been observed for asian Galerucinae species (Mohamedsaid 2004). Although no pattern is observed, some variation can occur in blocks, i. e., one modified antennomere occurs with one or two adjacent antennomeres also modified.

It seems that most, if not all, antennal variations could be regarded as the result of the presence of punctuation and setae in greater number, either for the production and/or reception of chemical compounds (i. e., pheromones) (Jolivet 2007). A study on *Diabrotica virgifera* Leconte, 1858, for instance, showed that male antennae have a much greater number of sensilla than females, and numerous glandular points linked to the production of chemical compounds allegedly to be attractive to females (Newman Jr et al. 1993). Unfortunately, there is little knowledge on the biology or even on the anatomy of Diabroticites to support this view as a more generalized tendency.

### Thorax

**Figure 6.**
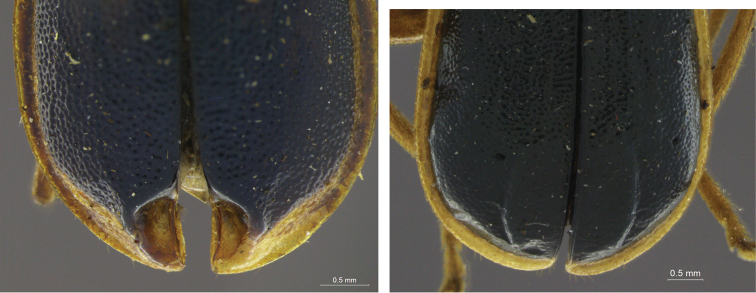
Detail of elytral modifications, left, *Isotes digna* (Gahan, 1891), male, right, *Paratriarius batesi* (Baly, 1859), male.

Although a great diversity of dimorphic characters occur in the thorax of several galerucines (Mohamedsaid and Furth 2011), features reported for Diabroticites are limited to elytra, and legs. In elytra, variation occurs only on the apical fifth, and are either depressions or callosities, both which can co-occur with punctuation (as in some species of *Isotes* – Figure 6, and in *Pseudodiabrotica* – the only genus supported on an elytral diagnostic character). Many species in genus *Paratriarius* show elytral dimorphic characters (such as the callosities present in *Paratriarius batesi*), and, although such features were never used to originally describe it, they have been used to support the definition of genus *Chanchamayia* Bechyné, 1956, now considered to be a subgenus in *Paratriarius* (Smith and Lawrence 1967, Wilcox 1972).

**Figure 7.**
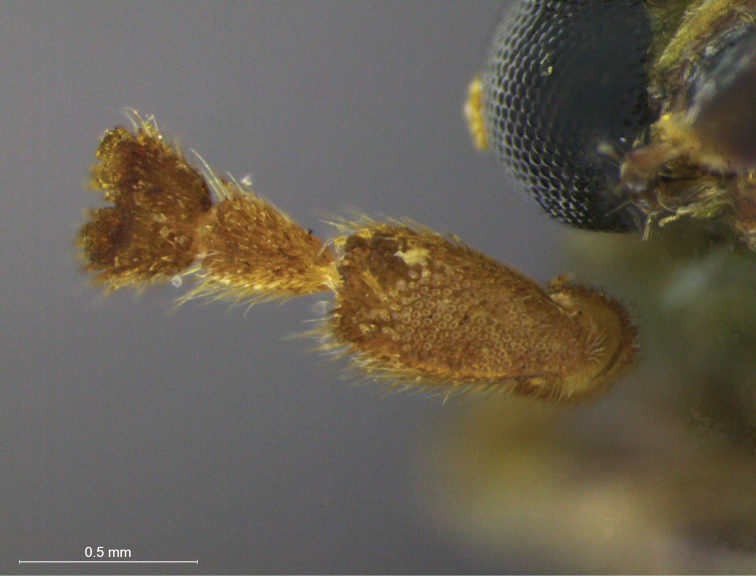
*Paranapiacaba prolongata* (Jacoby, 1882), detail of ventral surface of tarsomeres of prothoracic leg, male.

Only metathoracic legs lack alterations in male diabroticites. In accordance with the more general pattern, morphological differences in the pro- and mesothoracic legs usually are connected to the augmentation of absolute size in femora and tarsomeres I, the latter which are directly linked to the partial or total covering of the ventral surface by adhesive setae (Figure 7).

**Figure 8.**
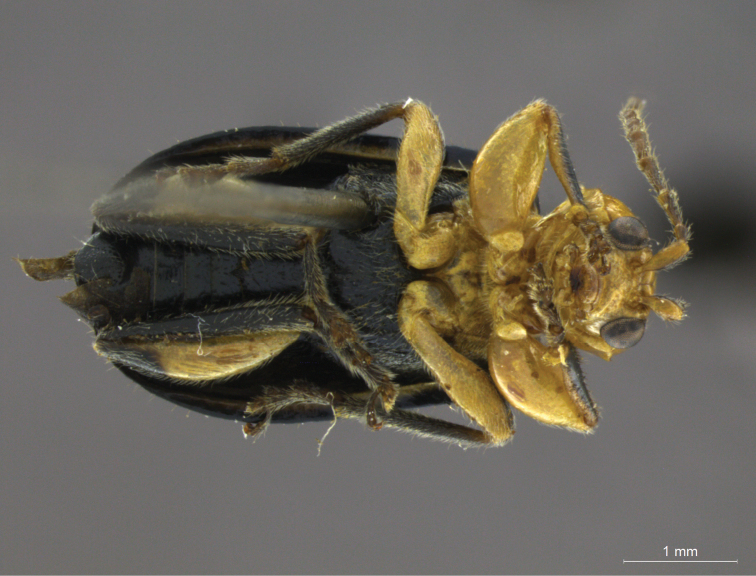
*Zischkaita serrana* Moura, 2003, ventral view, male.

**Figure 9.**
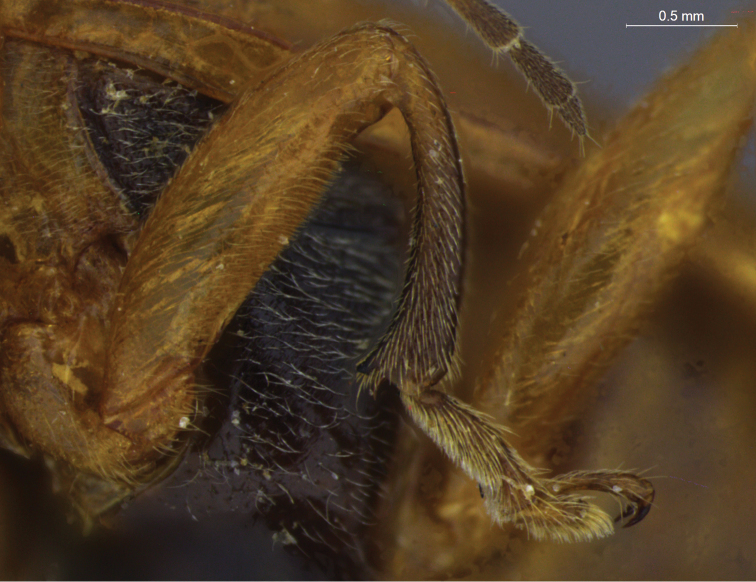
*Aristobrotica angulicollis* (Erichson, 1878), detail of mesothoracic leg, male.

Tibiae and femora can also be modified, being greatly enlarged (such as in *Zischkaita serrana* Moura, 2003 – Figure 8), and frequently with internal margins concave or bearing tubercles, forming the “prehensile organ” (Bechyné 1956). Bechyné’s concept of such structure is based on a combination of multiple adaptations and should be used with caution, since the homology of the “prehensile organs” can be difficult to assess. *Aristobrotica*, for instance, has been described as with one diagnostic feature: the “special build of the median tibiae in male”. The detailed analysis of species included, however, indications that there are at least two distinct types of “prehensile organs” being treated as the same modification. While type-species *Aristobrotica angulicollis* (Figure 9) bears only a concave mesotibiae with laterally flattened apex, *Aristobrotica mirapeua* Moura, 1997 and *Aristobrotica capillosa* Moura, 2011 (both illustrated in their original descriptions) display small projections of the ventral margin of the mesofemora (apical in *Aristobrotica mirapeua* and basal in *Aristobrotica capillosa*), and differently shaped tibiae (with a basal concavity in *Aristobrotica mirapeua* and slightly concave tibiae with apical flattening in *Aristobrotica capillosa*).

Moreover, general characters have been used to support definition of genera such as *Cornubrotica* and *Synbrotica*, a genus which is now a synonym of *Isotes*. The former was supposed to be distinguished by pro- and mesothoracic legs of males without emargination, which is rather common in the tribe, and the latter is characterized by antennomere III elongated and a “uniform pilosity covering the ventral surface of tarsomeres in both sexes” (freely translated from the original, in German) – something that does not accurately identify the males in this group, as they normally have distinctive adhesive disks in their tarsomeres.

### Abdomen

**Figure 10.**
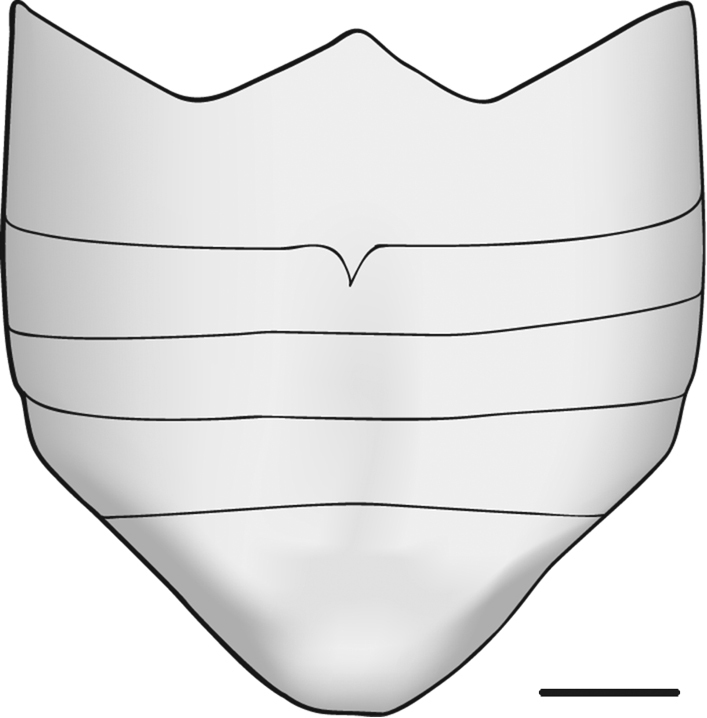
*Zischkaita serrana* Moura, 2003, schematic abdomen in ventral view, male. Scale bar = 0,5 mm.

The most common abdominal modification seen in some galerucines is the presence of processes with different shapes. Although no abdominal characters aid the definition of diabroticites genera, one character was observed for a single species in the group: a central triangular projection, postero-ventrally oriented, in the posterior margin of the ventrite I, in *Zischkaita serrana* (Figure 10). A similar alteration is observable in *Hemygascelis longicollis* Jacoby, 1896, an asian species that belongs to section Phyllobroticites in subtribe Luperina, a group thought to be a sister group of Diabroticina (illustrated in Mohamedsaid and Furth 2011).

## Conclusions

The taxonomic history of taxa in section Diabroticites, which dates to over 150 years of specimen sampling and species descriptions, has been supported primarily by scarcely detailed descriptions of morphological features, specially coloration and striking “sexual aberrations”. Sexually dimorphic characters have been the foundation for the creation of many genera in this group, albeit with vague definitions and a general disregard for comparative morphology. The use of inappropriate terminology by some authors has caused further confusion, since the habit of inferring homology from homonymy is common in the taxonomy of Chrysomelidae (Schmitt, 1996). This might have been the origin of the combination of many species into these scarcely defined genera in Diabroticites. As a result, most generic original definitions, based on dimorphic characteristics, are not correspondent to their actual assembly of species.

Nevertheless, the establishment of homology theories is far from being trivial, and some variations might even occur in patterns that can actually help the definition of certain taxa, although it is clear that many sexually dimorphic features found in this section are possibly singular and autapomorfic. In this case, a broader morphological study is necessary.

The comparison of the dimorphic characters in Diabroticites with their related taxa, such as the Asiatic Aulacophorites Chapuis, 1875 (Luperini) and remaining Galerucinae is desired, in order to understand the evolution of such characters. There are striking similarities among many modifications found in these groups and thus it should be useful in the evaluation of homology as well. Parallelism might be the more parsimonious choice in many cases, but that is yet to be tested. Understanding the morphology is critical for better character definitions. Also, genital characters, which have been generally ignored, should provide important characters, as well as the account of several non-dimorphic characters, usually regarded as too uninformative, without detailed consideration.

## Appendix

List of taxa with male specimens available in south-american collections.

*Acalymma albidovittatum* (Baly, 1889)

*Acalymma bivittulum* (Kirsch, 1883)

*Acalymma bruchii* (Bowditch, 1911)

*Acalymma carinipenne* (Bowditch, 1911)

*Acalymma exigua* Bechyné, 1958

*Acalymma granulipenne* (Bowditch, 1911)

*Acalymma incum* (Bowditch, 1911)

*Acalymma innubum* (Fabricius, 1775)

*Acalymma isogenum* Bechyné & Bechyné, 1968

*Acalymma punctatum* (Jacoby, 1887)

*Acalymma rubeolum* Bechyné, 1958

*Acalymma thiemei* (Baly, 1886)

*Acalymma vitigera* (Boheman, 1859)

*Acalymma vittatum* (Fabricius, 1775)

*Acalymma xanthographum* Bechyné, 1955

*Anisobrotica binisculpta* Bechyné & Bechyné, 1969

*Anisobrotica donckieri* (Baly, 1889)

*Anisobrotica nordenskiöldi* (Jacoby, 1907)

*Anisobrotica notaticollis* (Baly, 1889)

*Aristobrotica angulicollis* (Erichson, 1878)

*Aristobrotica capillosa* Moura, 2011

*Buckibrotica cinctipennis* (Baly, 1886)

*Cochabamba chacoensis* (Bowditch, 1911)

*Cochabamba chrysopleura* (Harold, 1875)

*Cochabamba diversicolor* (Baly, 1890)

*Cochabamba erythrodera* (Baly, 1879)

*Cochabamba marginata* (Harold, 1875)

*Cochabamba mera* Bechyné, 1956

*Cochabamba polychroma* Bechyné, 1956

*Cochabamba rugulosa* (Baly, 1886)

*Cochabamba variolosa* (Jacoby, 1878)

*Cochabamba volxemi* (Baly, 1889)

*Cornubrotica dilaticornis* (Baly, 1879)

*Cornubrotica iuba* Moura, 2005

*Diabrotica alegrensis* Bechyné & Bechyné, 1962

*Diabrotica amoena* (Dalman, 1823)

*Diabrotica antonietta* Bechyné, 1956

*Diabrotica aracatuba* Bechyné & Bechyné, 1964

*Diabrotica arcuata* Baly, 1859

*Diabrotica atrilineata* Baly, 1889

*Diabrotica atromaculata* Baly, 1889

*Diabrotica atrosignata* Baly, 1890

*Diabrotica boggianii* Bowditch, 1911

*Diabrotica chloropus* Harold, 1875

*Diabrotica clarki* Weise, 1916

*Diabrotica confraterna* Baly, 1889

*Diabrotica consentanea* Baly, 1886

*Diabrotica cryptochlora* Bechyné, 1956

*Diabrotica decaspila* Baly, 1890

*Diabrotica decempunctata* (Latreille, 1813)

*Diabrotica deliqua* Weise, 1921

*Diabrotica distincta* Jacoby, 1882

*Diabrotica egleri* Bechyné & Bechyné, 1961

*Diabrotica elata* (Fabricius, 1801)

*Diabrotica emorsitans* Baly, 1890

*Diabrotica enae* Marques, 1941

*Diabrotica fallenia* Bechyné, 1956

*Diabrotica flava* (Olivier, 1791)

*Diabrotica funerea* Bowditch, 1911

*Diabrotica fusibilis* Bechyné & Bechyné, 1970

*Diabrotica gracilenta* Erichson, 1847

*Diabrotica graminea* Baly, 1886

*Diabrotica kirbyi* Baly, 1890

*Diabrotica lamiina* Bechyné & Bechyné, 1969

*Diabrotica limitata* (Sahlberg, 1823)

*Diabrotica lutescens* Baly, 1890

*Diabrotica manaensis* Weise, 1921

*Diabrotica nitidicollis* Baly, 1889

*Diabrotica olivacea* Jacoby, 1882

*Diabrotica panchroma* Bechyné, 1955

*Diabrotica paranaensis* Marques, 1941

*Diabrotica pentazyga* Bechyné & Bechyné, 1970

*Diabrotica piceicornis* Baly, 1889

*Diabrotica piceosignata* Baly, 1890

*Diabrotica poecilenta* Bechyné, 1958

*Diabrotica propylaea* Bechyné & Bechyné, 1969

*Diabrotica quinquemaculata* (Fabricius, 1801)

*Diabrotica recki* Marques, 1941

*Diabrotica rufolimbata* Baly, 1879

*Diabrotica samouella* Bechyné, 1956

*Diabrotica schaufussi* Baly, 1890

*Diabrotica scripta* (Olivier, 1808)

*Diabrotica sedata* Baly, 1890

*Diabrotica serroazulensis* Bechyné & Bechyné, 1962

*Diabrotica sharpii* Kirsch, 1883

*Diabrotica sheba* Bechyné, 1958

*Diabrotica simulata* Baly, 1890

*Diabrotica sinuata* (Olivier, 1789)

*Diabrotica speciosa* (Germar, 1824)

*Diabrotica stenocoryna* Bechyné & Bechyné, 1970

*Diabrotica tarcisia* Bechyné, 1971

*Diabrotica tijuquensis* Marques, 1941

*Diabrotica transversa* Baly, 1890

*Diabrotica travassosi* Marques, 1941

*Diabrotica univittata* Jacoby, 1899

*Diabrotica viridans* Baly, 1889

*Diabrotica viridimaculata* Jacoby, 1878

*Diabrotica viridula* (Fabricius, 1801)

*Diabrotica wartensis* Cabrera & Sosa-Gómez, 2008

*Diabrotica westwoodi* Baly, 1889

*Ensiforma asteria* (Bechynné & Bechyné, 1962)

*Ensiforma caerulea* Jacoby, 1876

*Ensiforma chiquitoensis* (Bechyné, 1958)

*Gynandrobrotica caviceps* (Baly, 1889)

*Gynandrobrotica equestris* (Fabricius, 1787)

*Isotes agatha* (Bechyné & Bechyné, 1969)

*Isotes albidocincta* (Baly, 1889)

*Isotes bertonii* (Bowditch, 1912)

*Isotes bicincta* (Bowdtich, 1912)

*Isotes borrei* (Baly, 1889)

*Isotes brasiliensis* (Jacoby, 1888)

*Isotes cargona* (Bechyné, 1958)

*Isotes caryocara* (Bechyné, 1956)

*Isotes crucigera* (Weise, 1916)

*Isotes delicula* (Erichson, 1847)

*Isotes digna* (Gahan, 1891)

*Isotes eruptiva* (Bechyné, 1955)

*Isotes ignatia* (Bechyné, 1956)

*Isotes onira* (Bechyné & Bechyné, 1961)

*Isotes pollina* (Bechyné & Bechyné, 1962)

*Isotes puella* (Baly, 1886)

*Isotes sanguineipennis* (Baly, 1891)

*Isotes semiflava* (Germar, 1824)

*Isotes sibylla* (Bechyné & Bechyné, 1969)

*Isotes taeniolata* (Gahan, 1891)

*Isotes ternata* (Bechyné & Bechyné, 1961)

*Isotes valentina* (Bechyné, 1956)

*Isotes varipes* (Boheman, 1835)

*Paranapiacaba amplexa* (Erichson, 1847)

*Paranapiacaba biseriata* (Gahan, 1891)

*Paranapiacaba costalimai* (Marques, 1941)

*Paranapiacaba decemverrucata* (Gahan, 1891)

*Paranapiacaba diametralis* (Bechyné, 1956)

*Paranapiacaba melanospila* (Gahan, 1891)

*Paranapiacaba morretesi* Bechyné & Bechyné, 1969

*Paranapiacaba pereirai* Bechyné, 1958

*Paranapiacaba prolongata* (Jacoby, 1882)

*Paranapiacaba seraphina* (Bechyné, 1956)

*Paranapiacaba significata* (Gahan, 1891)

*Paranapiacaba subirregularis* (Bechyné & Bechyné, 1962)

*Paranapiacaba teinturieri* (Allard, 1894)

*Paratriarius batesi* (Baly, 1859)

*Paratriarius limbatipennis* (Baly, 1889)

*Platybrotica misionensis* Cabrera & Cabrera Walsh, 2004

*Zischkaita serrana* Moura, 2003
